# Short-time mentoring – enhancing female medical students’ intentions toward surgical careers

**DOI:** 10.1080/10872981.2024.2347767

**Published:** 2024-05-02

**Authors:** J. C. Mossanen, M. Schmidt, A. Brücken, M. Thommes, G. Marx, S. Sopka

**Affiliations:** a Department of Intensive and Intermediate Care, University Hospital RWTH Aachen, Aachen, Germany; b Department of Anesthesiology, University Hospital RWTH Aachen, Aachen, Germany; c AIXTRA – Competence Center for Training and Patient Safety, Medical Faculty, RWTH Aachen University, Aachen, Germany

**Keywords:** Female surgeons, role models, gender bias in surgery, short-time mentoring, surgical career

## Abstract

**Background:**

Women pursuing a career in surgery or related disciplines are still in the minority, despite the fact that women compose at least half of the medical student population in most Western countries. Thus, recruiting and retaining female surgeons remains an important challenge to meet the need for surgeons and increase the quality of care. The participations were female medical students between their third and fifth academic year. In this study, we applied the well-established psychological theory of planned behavior (TPB) which suggests that the intention to perform a behavior (e.g. pursuing a career in surgery) is the most critical and immediate predictor of performing the behavior. We investigated whether a two-part short-mentoring seminar significantly increases students’ intention to pursue a career in a surgical or related specialty after graduation.

**Method:**

The mentoring and role-model seminar was conducted at 2 days for 90 minutes by six inspiring female role models with a remarkable career in surgical or related disciplines. Participants (*N* = 57) filled in an online survey before (T0) and after the seminar (T1). A pre-post comparison of central TPB concept attitude towards the behavior, 2) occupational self-efficacy and 3) social norm) was conducted using a paired sampled t-test. A follow-up survey was administered 12 months later (T2).

**Results:**

The mentoring seminar positively impacted female students’ attitude towards a career in a surgical specialty. Female students reported a significantly increased positive attitude (*p* < .001) and significantly higher self-efficacy expectations (*p* < .001) towards a surgical career after participating in the mentoring seminar. Regarding their career intention after the seminar, female students declared a significantly higher intention to pursue a career in a surgical specialty after graduating (*p* < .001) and this effect seems to be sustainable after 1 year.

**Conclusion:**

For the first time we could show that short-mentoring and demonstrating role models in a seminar surrounding has a significant impact on female medical student decision´s to pursue a career in a surgery speciality. This concept may be a practical and efficient concept to refine the gender disparity in surgery and related disciplines.

## Introduction

Despite the fact that female students compose more than half of current medical school classes, women are a minority in surgical specialties [1]. According to data from the Association of American Medical Colleges (AAMC) in 2017 women make up less than 25% within 10 surgical specialities and in 2021 just 32 of 352 chairs of surgery departments in the United States were hold by women. This gender gap in surgical specialities is visible in all countries developed and developing countries although especially in western countries, more women complete medical studies than men [2–5].

In recent years, gender inequality in medicine has drawn attention and research has identified several reasons for this still existing disparity, such as work-life balance, lack of support for pregnancy and parenting, lack of mentorship and role models as well as a culture which is not beneficial for women [6,7]. During their practice in surgical specialities, female medical students undergo rotations and trainings in a non-conducive work environment, as exemplified by sexual discrimination or gender being a barrier to career promotion. Thereby, female role models in these fields are disproportionately affected by depression, burnout and a lack of estimation [8,9]. Previous studies identified mentorship and role models as important factors in order to recruit medical students for certain specialities. The Review from Schmidt et al. from 2016 included 12 studies regarding mentoring in surgical specialties and described good mentors and role models are integral to students` decision to pursue a career in surgery [10]. In these different studies mentoring took place in preclinical surgical elective, with faculty mentors during the general surgery education and in the operating room. The authors highlighted good mentors as the truth factor influencing students to pursue a career in surgery. Due to the existing gender bias, female mentors or role models are rare in surgical residencies and at the same time studies revealed that women prefer gender-concordant mentoring [11]. Studies in various countries that have examined the role of mentoring for aspiring female surgeons constantly show more female surgeons when mentoring is offered by female surgeons [12–16]. In a survey 80% of female members of the Royal College of Physicians and Surgeons in Canada indicated the need for a female mentor [17]. The limiting factor for mentorship and role models is the number of women needed to give plenty students and residents insights in their working life and one by one mentoring. For this reason, we developed the concept of short-time mentoring.

However, with ongoing efforts there is still potential for major improvement to increase the attractiveness of surgical disciplines for female medical students. New data highlight the importance of reducing gender bias in surgery. In a large population of 1.3 million female and male patients in Canada a sex discordance between surgeon and patient was associated with an increased likelihood of adverse outcomes. This association was observed for female patients treated by male physicians without a corresponding result among male patients treated by female physicians [18]. Therefore, the aim of our study was to investigate whether a new concept of short-time mentoring could strengthen the intention of female medical students to pursue a career in surgical or related disciplines. For this purpose, female role models and mentorship from different surgical specialties as well as intensive care medicine were offered during a seminar. We built this study on the well-established psychological theory of planned behaviour (TPB) [19]. The TPB framework has been applied in various settings and has demonstrated high predictive validity of intention and behavior throughout, e.g., career and academic intentions or health-related behavior (e.g [20–22]). TPB directly links beliefs to behavior and suggests that the *intention* to perform a behavior (e.g., pursuing a career in surgery) is the most critical and immediate predictor of actually performing the behavior [19]. Several empirical studies and meta-analyses support this notion (e.g [23,24]). Behavioral intention itself is predicted by three central factors: 1) attitude toward the behavior, 2) occupational self-efficacy and 3) social norm. Applying TPB to predict female students’ career aspirations in surgery or related disciplines, *attitudes* refer to students’ evaluation regarding a career in surgery and its consequences (e.g., favorable or unfavorable), occupational *self-efficacy* refers to female students’ beliefs in their capacity to fulfill work-related tasks or activities in a surgical discipline, and *subjective norm* refers to their expectation that significant others (e.g., partner, friends, family members) approve of a career in surgery (i.e., perceived social pressure).

To this date, research on attitudes, self-efficacy and norms specifically related to female medical students pursuing a career in surgery is scarce. However, results by Hill et al.. (2014) who qualitatively explored perceptions of medical students towards surgeons and surgical careers imply that strong stereotypes are predominant [25]. These stereotypes included perceptions of surgeons being confident and intimidating and the surgical culture as competitive and masculine.

The mentoring seminar aims to specifically target female students’ attitude towards a career in surgery as well as their self-efficacy beliefs to succeed in a surgical discipline through an open dialogue with female surgical role models. Previous research has shown that positive encounters with role models can influence perceptions of a career in surgery (e.g [26]). While mentoring is recognized as a pivotal factor in career choice, the impact of short-time, group-based mentoring on female medical students’ career intentions in surgery remains underexplored. The central aim of this research therefore is to investigate whether the two-part mentoring seminar can strengthen female students’ surgical career intention and positively impacts self-reported central TPB concepts.

## Methods

### Ethics

Ethical approval (Ethics Review Board document 21–429) was granted according to the ethical principles of the World Medical Association’s Declaration of Helsinki [27] on 29 November 2021 by the Institutional Ethics Review Board of the University Hospital, RWTH Aachen.

### Study design

**Figure 1.**
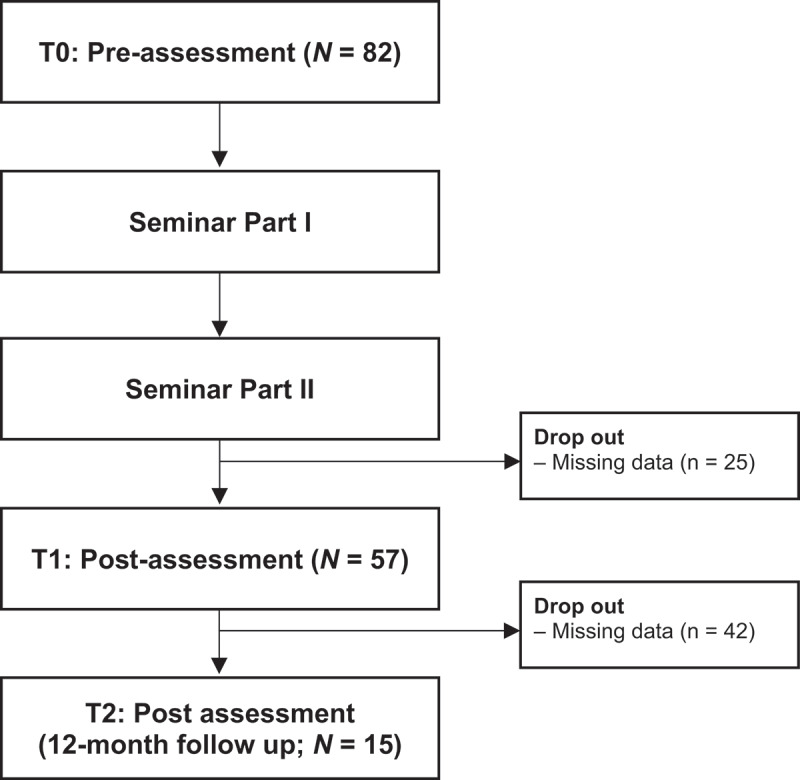
Study flow chart.

The present study follows a one-group pretest-posttest design in which participants were asked to fill out an online-survey before (T0) and after attending the mentoring seminar (T1). Both participation in the seminar and in the study were voluntary. Solely anonymous data were collected. A follow-up survey was administered 12 months later (T2). Data collection was carried out during four seminars held between November 2021 and May 2023. All participants of the seminar were included in the study sample. No exclusion criteria were defined. Figure 1 depicts a flow chart of the study.

### Sample size and selection

Sample size planning for a two-tailed paired sample t-test was performed with G*Power. A medium effect size dz of 0.5, an α significance level of .05 and a power (1-β) of 90% resulted in a required sample size of *N* = 54. Students interested in the seminar had to be at least in their 5^th^ semester in order to attend the seminar. Seminar places were allocated on a first come first serve basis. In addition, there was a waiting list for interested students that were contacted if participants canceled their registration.

### Participants

From a total of 82 participants, *n* = 57 were included in further analysis with a mean age of 23.11 years (SD 3.64). On average, students were in the fourth year of their medical study (7^th^ semester, SD 2.41 study semesters). As the mentoring seminar was specifically advertised to female students with a general interest in surgical subjects, 28 participants (50.9%) reported to already have famulated in a surgical specialty during their studies and 12 students (21.4%) have started their PhD trajectory in a surgical specialty.

### Seminar concept

The seminar ‘Exciting – but not feasible as a woman?’ (Original German version ‘Spannend – aber als Frau nicht machbar?’) was newly established in 2021 and offered to female medical students of the medical faculty at RWTH Aachen University with the intention to increase the attractiveness of surgical specialities and related disciplines. Female students from 5^th^ to 11^th^ medical school classes (semester) were invited to participate. Core elements of the seminar were inspiring female role models with a remarkable career in surgical or related disciplines providing insights into their career path including potential obstacles they faced as well as conducive factors that supported their career decisions. Mentoring took place in small groups, consisting of one mentor and five students each. The seminar was divided into two parts of 1.5 hours each. In the first seminar students could ask questions in plenum while in the second seminar they could choose the mentor in which they were specifically interested in due to her specialty or personality. In both parts participants were given the chance to ask questions related to any part of interest regarding the career steps, work-life balance, maternity leave, supporters and personal assessments about work atmosphere in surgery specialities or related discipline.

### Mentor selection

Mentors were all female surgeons and intensive care physicians from different surgical disciplines. Mentors were all experienced specialists or senior physicians in their disciplines with at least 10 years of clinical experience. The personal background and mapping paths of the female mentors deliberately presented different career paths. Mentors with and without children, research or experiences broad were presented.

## Measures

### Demographics

Demographic items included age, gender, semester as well as interest and experience in surgical or related disciplines. Further, participants were asked about their interest in or already completed activities (e.g., internship, clerkship, practical year, dissertation) in a surgical or related discipline.

### TPB concepts

Measures for central TPB concepts were constructed following Ajzen’s (2002) recommendations [28] (Appendix A). Before TPB items were presented to participants, a short introductory text was presented to students: “All following items have to do with your opinions and ideas regarding a career in a surgical specialty, including surgical intensive care, general surgery, thoracic and cardiac surgery, vascular surgery, neurosurgery and trauma surgery. Responses were given on a 7-point Likert scale.

### Attitude

A two-item measure was constructed to assess students’ attitude towards a career in a surgical specialty. The items consisted of the introductory sentence ‘I generally regard the job in a surgical specialty as’ and participants were subsequently asked to rate the items on a 7-point semantic scale on undesirable/desirable and unexciting/exciting. This measurement approach has been suggested by Ajzen (2002) and has been applied in previous research (e.g [21].

### Self-efficacy

Self-efficacy regarding a career in a surgical specialty was assessed using the validated German 5-item scale for measuring occupational self-efficacy scale (BSW-5-Rev) by Knispel et al.. (2021) [29]. An example item is”I know that I can fulfill the job requirements if I only want to”.

### Subjective norm

Subjective norm was assessed by a three-item measure regarding a career in a surgical specialty following Ajzen’s (2002) recommendations [28]. Example items include: ‘Most of my significant others believe I should start a job in a surgical specialty within the foreseeable future after I finish my degree’ and ‘most of my significant others would support me taking a job in a surgical specialty within the foreseeable future after I finish my degree’.

### Career intention

The intention to pursue a career in a surgical specialty after graduation was assessed with a three-item measure. An example item is ‘After graduation I intent to pursue a career in a surgical specialty’. Answers were given on a 7-point Likert Scale ranging from 1 (does not apply to me) to 7 (fully applies to me).

### Perceived external obstacles

In order to assess perceived external obstacles, open-ended items were developed in-house. Example item ‘The many obstacles on the path to employment in a surgical or related discipline would prevent me from entering such employment within the foreseeable future after completing my degree.’. Answers were given on a 7-point Likert scale ranging from 1 = very unlikely to 7 = very likely.

### Statistical analysis

Data analysis was performed using IBM SPSS Statistics, Version 28 (IBM Corp., Armonk, NY, USA). A paired sample t-test was used to determine mean differences between pre- and post-assessment of the previously described measures.

### Hypothesis

We hypothesize that the mentoring seminar has a significant positive impact on female medical students’ ratings of attitude, self-efficacy, subjective norm and career intention towards pursuing a career in a surgical specialty.

## Results

### Descriptive statistics and intercorrelations between TPB scales

Table 1 shows descriptive statistics (means and standard deviations), scale reliabilities (Cronbach’s α) and intercorrelations between TPB scales.

**Table 1.** Descriptives, Cronbach’s α and intercorrelations between study scales.

Variable	*n*	*M*	*SD*	*Cronbach’s α*	1	2	3	4	5	6	7	8
1. Attitude_T0_	46	6.10	0.98	.74	-							
2. Self-efficacy_T0_	45	4.60	0.95	.76	.36*	-						
3. Subjective norm_T0_	46	4.70	1.27	.65	.47**	.53**	-					
4. Career intention_T0_	46	5.19	1.52	.97	.82**	.46**	.45**	-				
5. Attitude_T1_	44	6.40	0.74	.76	.67**	.35*	.27	.72**	-			
6. Self-efficacy_T1_	45	5.31	0.75	.74	.33*	.65**	.54**	.40**	.50**	-		
7. Subjective norm_T1_	46	4.89	1.14	.65	.49**	.38*	.72**	.49**	.34*	.56**	-	
8. Career intention_T1_	46	5.80	1.21	.96	.68**	.41.**	.37*	.73**	.89**	.54**	.46**	-

Note. ** = *p* < .01, * = *p* < .05.

Descriptive statistics show a general tendency of an increased mean at T1 with the highest increase for the variable self-efficacy. Cronbach’s α represents the extent to which a scale is internally consistent and ranges from .65 for subjective norm to .97 for career intention. In terms of career intention, this suggests that items are excellent in terms of internal consistency but may show redundancies whereas for subjective norm the items might not represent the construct sufficiently. Cronbach’s α value for attitude and self-efficacy are on an acceptable level. Furthermore, intercorrelations between the study scales show significant positive correlations with the greatest correlation of attitude and career intention.

### Statistical analyses: pre-post comparisons

To investigate whether the mentoring seminar significantly influences central TPB constructs in the expected direction, mean difference before and after the mentoring seminar were analyzed to assess the impact of the seminar on relevant outcome measures. Table 2 presents the pre-post comparisons of a paired sample t-test.

**Table 2.** Pre-post comparison of key TPB constructs of pursuing a career in a surgical specialty before and after the mentoring seminar.

TPB constructs	n_pre_	M_pre_	SD_pre_	n_post_	M_post_	SD_post_	t	p	d
Attitude	55	6.05	0.99	55	6.39	0.77	−3.73	<.001	.38
Self-efficacy	55	4.65	0.91	55	5.34	0.75	−7.31	<.001	.94
Social norm	57	4.75	1.25	57	4.87	1.20	−1.05	.299	.10
Career intention	57	5.16	1.51	57	5.72	1.27	−4.28	<.001	.40

Notes. TPB = Theory of planned behavior.

As can be inferred from Table 2, and in line with expectations, the mentoring seminar positively impacted female students’ attitude towards a career in a surgical specialty. Specifically, students reported a more positive attitude towards a surgical career after participating in the mentoring seminar in comparison to before their participation. Similarly, they reported significantly higher self-efficacy expectations towards a surgical career after participating in the mentoring seminar. However, results showed no significant difference between students’ self-reported social norm (i.e., the perception that significant others would approve of a career in a surgical specialty) before and after the seminar.

**Figure 2.**
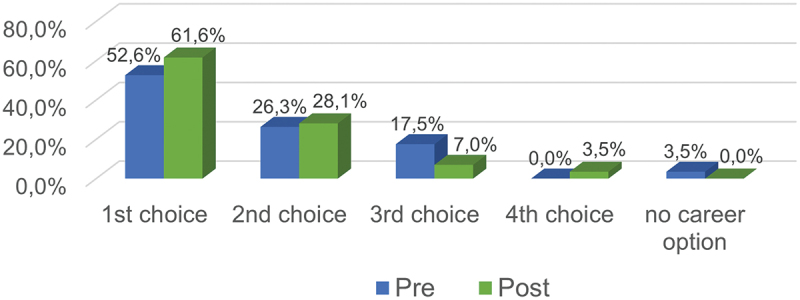
Pre-post comparison of female medical students' surgical career preference before and after the mentoring seminar.

Regarding their career intention, female medical students reported a significantly higher intention to pursue a career in a surgical specialty after graduation after participating in the mentoring seminar. These results are also reflected in the reported surgical career preferences as shown in Figure 2.

Additionally, as can be inferred from Figure 2, students are more likely to indicate a surgical career as their first career choice. Additionally, the percentage of students for whom a surgical career was no career option decreased after the seminar. Results of a Wilcoxon signed-rank test reveals a significant pre-post-difference in the number of female medical students reporting a surgical career to be their favored career option (Z = −2.98; *p* = 0.003).

### Descriptive qualitative feedback

Additionally, we received some qualitative feedback on what the students especially liked about the seminar and what they would suggest for improvement in the future. Twenty-one students positively mentioned that the communication with the physicians was very open and questions were answered very honestly. Four students mentioned that they especially liked the small group work and two students positively mentioned the representation of various specialties. Three students would have liked the seminar to be longer in order to ask even more questions. One student would have liked to see more role models from surgical specialties which is a challenge to tackle for future seminars as role models are very rare.

### Supplemental analyses (T2)

The 12-month follow-up online survey (*n* = 15) seems to support the positive impact of the mentoring seminar on student decision for a surgery specialty. 73.3% of the participants reported a surgical career as their favored career choice and indicated that the mentoring seminar was an important reason for this decision. This data points out that the impact of the seminar and the positive evaluation may be permanent.

## Discussion

The seminar was designed for female students interested in a career in surgery. Resulting from this, the initial percentage of female students preferring a surgical career as first choice was comparable high with 52.6%. Nevertheless, the mentoring seminar could even increase this percentage significantly up to 61.4% (*p* = 0.003). Our first 1-year follow-up shows similar percentage of students declaring a surgical career as their first choice (73.3%) what might indicate a long-standing effect of the mentoring and role model seminar.

In terms of the TPB record, we could not see any differences regarding the student expectation that significant others approve of a career in surgery (subjective norm) comparing the results before and after the mentoring seminar. These results were as expected since the focus of the seminar was on the attitude towards the behavior of female medical students and their occupational self-efficacy. No effects on social norm can be explained in the way that significant others were neither affected nor addressed by the seminar.

A study from 2022 also investigated the question if ‘Same-Gender Speed-Mentoring’ changes women´s perception of a career in surgery. The authors could show that a 90 minutes speed dating with female surgeons of different specialties is an effective intervention for addressing negative perceptions of a surgical career for female surgeons [30]. Our study focused on TPB constructs (attitude, self-efficacy, subjective norm and career intention) and could show for the first time a significant increase in female students’ intention to pursue a career in surgery after participating in the short-mentoring. Moreover, female students reported a more positive attitude and higher self-efficacy expectations towards a surgical career after the short-mentoring seminar. These findings thereby extend previous research by providing more nuanced insights into *why* short-time mentoring positively impacts female students surgical career intentions. In their review about the experiences of female surgeons around the world including 139 studies from 26 countries, Xepoleas and colleagues (2020) identified the lack mentorship as career barrier and it seems to be important to establish a critical mass of woman in surgery to encourage female students to go to surgical disciplines [12].

Compared to males, females in surgery face several challenges such as sexual harassment, training related issues (decreased autonomy, increased burnout and depression), lack of mentor- and sponsorship, lack of diversity in institutional, professional and editorial positions and inequality pay [31]. Additionally, there are often inappropriate policies for maternity leave and a lack of knowledge regarding maternity leave rights [32].

Female medical students closely interact with residents during their rotations and trainings in surgery and are confronted with a female dismissive environment due to the mentioned reasons. Studies could show that the recruitment of female students into a surgical specialty is possible and successful via mentoring and role models [10] but due to the lack of female surgeons, mentors and role models remain scarce [33]. Furthermore, Carapinha et al.. (2016) could show that women prefer a gender-concordant mentoring [11].

With high statistical significance (*p* < 0.001) a study from the US found that a higher proportion of female medical students pursued surgery if more female surgical role models are visible. Another Study from Australia and New Zealand demonstrated that the absence of interactions with other women was a reason why female trainees left surgical training [34,35]. In our short mentoring, we focused on these problems for women in surgery. One possible solution to the barrier for women in surgery is to increase the mentorship and visibility of woman in these specialties. This effect has been demonstrated in the US and female surgeons in different countries identified the lack of especially female mentorship as a barrier of career advancement [34,36–39]. All these studies showed the positive effect of mentorship but there is no solution to resolve the disparity between existing female mentors and the actual need for female mentors. Our data suggested that a short mentoring concept could be a possible solution to this problem.

Many questions of the female students referred to the female dismissive environment, maternity leave and sexual harassment. The results for the increase of self-efficacy clearly show the positive impact of good role models with helpful advices and experiences. The participants reported about the positive impact of inspiring and authentic woman as an important example for convincing career paths in surgical disciplines.

It is also known that interventions like mentoring result in increased promotion of women [40,41]. In our mentoring seminar, we synergize these approaches. Applying TPB, we showed that the mentoring seminar positively impacted female students’ attitude and self-efficacy expectation towards a surgical career. At the same time, the expenditure of time for mentors is manageable. As such, his approach solves the predominant problem that female faculty mentors are limited in many surgical specialties and senior physicians are hardly to convince to mentor students for an extended time [33]. Our concept opens the possibility to present different career paths, personalities and surgical disciplines in a short-time period. Thereby the mentoring addressed every student in another way. Furthermore, our study and the results display that the TPB is very well applicable for the context of medical education and can be of great use in the future in order to investigate and medical students’ career intentions.

In 2020 an interventional study could show that negative stereotypes about women and a pro-male gender bias affect the commitment and technical performances of female and male individuals pursuing careers in a surgical specialty [42]. Programs for same-gender mentoring might change the factors contributing to underrepresentation of women in surgery as well as a pro-male bias and stereotypes. This might consequently also lead to positive effects concerning patients care [18]. These positive effects can be observed after the same gender short-time mentoring and were confirmed by comments of the female students highlighting the mentoring only by women. Even the different age of the mentors was important to the students not only the different disciplines. Our findings encourage us to think mentoring in a new way with more mentors and mentees for a short time to show different ways and aspects of surgical disciplines and share more experiences with more people. Short mentoring should be an option in many areas with too few same-sex mentors and in the medical field it should not be limited to surgical specialties.

## Limitations

The limitation of this study includes the restricted number of participants after four mentoring seminars. We decided to define a maximum number of participants for each seminar to give every student the possibility to ask all their questions. Another limitation is the individuality of mentoring, which initially depends on the respective mentors. It would be necessary to carry out the concept at other universities and with other mentors in the future to prove an independent effect of short mentoring. The 12-months later data are only available for a cohort of a single year which limits the generalizability of our findings about longstanding long-term effects after the mentoring seminar. Furthermore, the follow-up survey focused on the career intention and the actual behavior of the students because an assessment of attitude, self-efficacy and subjective norm could not be attributed to the mentoring seminar with certainty. Therefore, it is not possible to derive interpretations about differences between regarding T2 concerning these variables as the informative value of these results would have been limited. It cannot be ruled out that other factors influence the student choice in the period after the short mentoring, which cannot be captured by our survey. Moreover, since the participation in the mentoring seminar as well as in the survey was voluntary, high dropout rates lead to missing data which should be taken into account when interpreting the data. Finally, this is a monocentric study and we intend to establish the seminar in other universities and in a virtual version in order to give more students access to Short-mentoring and to gain multicenter data and compare the effects of personal and virtual mentoring.

## Conclusion

Our short-time mentoring seminar for female medical students leads to significant and sustainable increase in their attitude, self-efficacy and career intentions to focus on a surgical or related discipline. The novelty of this mentoring form is that it creates an opportunity to permit more female students mentoring and meeting role models without overshooting the amount and the time of female mentors in surgery. By doing so, it actively addresses the ongoing gender bias in surgery and offers the opportunity for replication in order to spread these positive effects more widely.

## Data Availability

Study material and datasets are available from the corresponding author upon reasonable request.

## Appendix A. Original items [German language]

**Table:**

Theorie des geplanten Verhaltens (zentrale Variablen)
**Einstellung**
Die berufliche Tätigkeit in einem chirurgischen Fach empfinde ich insgesamt als erstrebenswert.
Die berufliche Tätigkeit in einem chirurgischen Fach empfinde ich insgesamt als spannend.
**Selbstwirksamkeit**
Ich weiß genau, dass ich die an den Beruf gestellten Anforderungen erfüllen kann, wenn ich nur will. Ich weiß, dass ich die für den Beruf erforderlichen Fähigkeiten wirklich habe.
Ich weiß, dass ich genügend Interesse für alle mit dem Beruf verbundenen Anforderungen habe.
Schwierigkeiten in dem Beruf sehe ich gelassen entgegen, da ich meinen Fähigkeiten vertrauen kann.
Es bereitet mir keine Schwierigkeiten, meine beruflichen Absichten und Ziele zu verwirklichen
**Soziale Norm**
Die meisten meiner Bezugspersonen glauben, ich sollte innerhalb absehbarer Zeit nach Beendigung meines Studiums eine Tätigkeit in einem chirurgischen Fach aufnehmen.
Die meisten meiner Bezugspersonen würden es unterstützen, wenn ich innerhalb absehbarer Zeit nach Beendigung meines Studiums eine Tätigkeit in einem chirurgischen Fach.
Wenn ich innerhalb absehbarer Zeit nach Beendigung meines Studiums eine Tätigkeit in einem chirurgischen/angrenzenden Fach aufnähme, so fänden das die meisten meiner Bezugspersonen.
**Karriereintention**
Wie groß ist Ihre Absicht innerhalb absehbarer Zeit nach Beendigung Ihres Studiums eine Tätigkeit in einem chirurgischen Fach aufnehmen?
Wie sehr wollen sie versuchen innerhalb absehbarer Zeit nach Beendigung Ihres Studiums eine Tätigkeit in einem chirurgischen Fach aufnehmen? Wie stark sind Sie entschlossen innerhalb absehbarer Zeit nach Beendigung Ihres Studiums eine Tätigkeit in einem chirurgischen Fach aufnehmen?
